# Obesity Is an Independent Prognostic Factor That Reduced Pathological Complete Response in Operable Breast Cancer Patients

**DOI:** 10.3390/medicina60121953

**Published:** 2024-11-27

**Authors:** Murad Guliyev, Özkan Alan, Murat Günaltılı, Shamkhal Safarov, Mehmet Cem Fidan, Gülin Alkan Şen, Ezgi Değerli, Berrin Papila, Nebi Serkan Demirci, Çiğdem Papila

**Affiliations:** 1 Division of Medical Oncology, Department of Internal Medicine, Cerrahpaşa Faculty of Medicine, İstanbul University-Cerrahpaşa, İstanbul 34098, Turkey; ozkan.alan@hotmail.com (Ö.A.); muratgunaltili@hotmail.com (M.G.); shamxalnn@gmail.com (S.S.); mcemfidan@hotmail.com (M.C.F.); gulinalkan@msn.com (G.A.Ş.); ezgitastan.19@gmail.com (E.D.); drserkannebi@yahoo.com (N.S.D.); bpapila@iuc.edu.tr (Ç.P.); 2 Department of General Surgery, Cerrahpaşa Faculty of Medicine, İstanbul University-Cerrahpaşa, İstanbul 34098, Turkey; berrin.papila@iuc.edu.tr

**Keywords:** breast cancer, obesity, body mass index, pathological complete response, neoadjuvant treatment

## Abstract

*Background and Objectives*: Obesity is a significant risk factor for the development of breast cancer (BC) and associated poorer outcomes. A pathological complete response (pCR) with neoadjuvant chemotherapy (NACT) correlates with improved long-term prognosis in BC patients. In this study, we aimed to investigate the predictive effect of obesity on achieving pCR following NACT. *Methods*: This single-center retrospective study included patients with operable BC who were treated with NACT. Patients were categorized based on their pre-chemotherapy body mass index (BMI), including non-obese (<30 kg/m^2^) and obese (≥30 kg/m^2^) groups, and pathological responses to NACT were compared. *Results*: A total of 191 female patients were included in this study; of these, 83 (43.4%) were obese and 108 (56.6%) were in the non-obese group. Obesity was more common in postmenopausal patients, and the median age of obese patients was significantly higher compared to non-obese patients. Patients in the obese group demonstrated significantly lower pCR rates compared to the non-obese group (30% vs. 45%, *p* = 0.03). The histological subtype assessment indicated that only in the HR-positive/HER2-negative patients was the pCR rate significantly lower in the obese group compared to the non-obese group (11% vs. 27%, *p* = 0.05). According to menopausal assessment, a significant difference in pCR rates was observed only among postmenopausal patients, with rates of 29% in the obese group compared to 52% in the non-obese group (*p* = 0.03). In logistic regression analysis, obesity (OR: 0.52, 95% CI: 0.28–0.97; *p* = 0.04) and a low Ki-67 score (HR: 2.7, 95% CI: 1.37–5.53; *p* = 0.003) were independently associated with a decreased rate of pCR. *Conclusions*: The impact of obesity on achieving pCR in BC patients undergoing NACT remains controversial. Our study revealed that obesity was an independently significant negative predictive factor for achieving pCR.

## 1. Introduction

According to the current cancer database, breast cancer was the second most common type of cancer in the world behind lung cancer and constituted 11.6% of all new cancer cases in 2022 [1]. The incidence rate of breast cancer in women is steadily increasing each year [2]. Regardless of the decrease in mortality rates associated with breakthroughs in cancer screening and treatment modalities [3], breast cancer remains the leading cause of cancer-related death among women.

Obesity is one of the most common public health problems worldwide, and the age-standardized prevalence progressively increased in 90% of countries worldwide from 1990 to 2022 [4]. The global prevalence of obesity has been on an upward trajectory, and its potential contribution to cancer treatment outcomes is a developing concern that necessitates additional investigation. Observational studies have found an association between being overweight and the prevalence of endometrial, ovarian, and breast cancer, with a high BMI having a detrimental effect on both cancer development and outcomes [5,6]. Obesity is a well-known risk factor for the development of breast cancer, especially in postmenopausal women [7,8]. Moreover, obesity is independently related to poor breast cancer outcomes and is also correlated with an advanced stage of breast cancer at the time of diagnosis [9,10]. Several factors have been hypothesized for clarifying this relationship, including the synthesis of pro-inflammatory cytokines by adipose tissue (particularly visceral fat), elevated levels of estrodiol, and insulin resistance [11,12].

Chemotherapy is a crucial treatment component to prevent recurrence for early-stage (I–III), higher-risk BC. Neoadjuvant chemotherapy (NACT) is intended to downstage the extent of disease in the breast and regional lymph nodes. Downstaging may enable less extensive surgery, including breast-conserving surgery in replacement of mastectomy, thus enhancing cosmetic outcomes and decreasing postoperative complications like lymphedema [13,14]. The achievement of pathological complete response (pCR) after NACT in the breast and axilla is an important predictor of long-term outcomes. A meta-analysis of 11,955 participants from 12 randomized clinical trials collected from Collaborative Trials in Neoadjuvant Breast Cancer (CTNeoBC), demonstrated long-term benefit on survival for patients achieving pCR [15].

The influence of obesity on achieving pCR in operable breast cancer patients who undergo NACT is still a topic of debate. Obesity is associated with decreased pCR rates in meta-analyses in the literature, whereas various other investigations have not found any significant relationship [16,17,18,19]. NACT facilitates the evaluation of the potential correlation between obesity and the in vivo response to chemotherapy. Following the NACT, evaluating the relationship between obesity and pCR allows for a more precise understanding of obesity’s impact on breast cancer outcomes.

The purpose of this study was to investigate the impact of obesity on pathological complete response rates in early/locally advanced stage, operable breast cancer patients after NACT.

## 2. Materials and Methods

### 2.1. Patients 

Women aged 18 years or older with a diagnosis of BC and treated with neoadjuvant therapy between 2012 and 2023 in the Medical Oncology Clinic of Cerrahpaşa Faculty of Medicine were evaluated retrospectively. Baseline data were extracted from databases and medical records. This study included patients with histopathologically confirmed BC, radiologically proven early/locally advanced stage disease, and undergoing surgery after neoadjuvant treatment. We excluded patients without follow-up data and patients who received neoadjuvant therapy in the different hospitals.

All study patients underwent a BC diagnosis through core biopsy or fine needle aspiration, with tissue evaluations conducted by a specialized breast pathologist prior to the initiation of NACT. A multi-disciplinary tumor board made all treatment decisions, strictly following international and national guidelines. NACT was administered to patients based on their risk, determined by clinical and histopathological parameters. The status of estrogen receptor (ER), progesterone receptor (PR), and human epidermal growth receptor 2 (HER2) was assessed by the American Society of Clinical Oncology/College of American Pathologists (ASCO/CAP) guidelines [20], and BC subtypes were categorized as hormone receptor (HR)+/HER2−, HR+/HER2+, HR−/HER2+, and triple negative (TNBC, HR−/HER2−). HR+/HER2− patients were evaluated as Luminal-A and Luminal-B groups according to the St. Gallen International Expert Consensus [21]. Body mass index (BMI) was calculated as pre-chemotherapy weight (kg) divided by square of height (m^2^), and patients were classified into two groups as non-obese (BMI < 30 kg/m^2^) and obese (BMI ≥ 30 kg/m^2^) based on World Health Organization criteria [22].

Age at diagnosis, menopausal status, histological features of tumor, hormone levels, tumor subtypes, tumor stage, NACT regimens, and pathological response rate to NACT were evaluated. Pathological complete response is defined as the absence of residual invasive cancer in breast surgical specimens (ypT0/is) and the absence of tumor cells in the axillary lymph nodes (ypN0) [15].

This study was approved by the local ethics committee for clinical trials (date: 17 April 2024 and number: E-83045809-804.01-96734), and the need for informed consent was waived because of the retrospective nature of this study. The whole procedure and steps of the study were executed in accordance with the ethical standards indicated in the Declaration of Helsinki.

### 2.2. Statistical Analysis

Statistical analyses were conducted using SPSS version 26. Stratification was performed according to the body mass index. We analyzed the data using conventional descriptive statistics, which included the mean, standard deviation, median, and range for continuous variables, as well as the frequency and proportion for categorical variables. The characteristics of the patients were compared with the Fisher or Chi-squared test for categorical data and a t-test for continuous data. Univariate and multivariate logistic regression models were conducted to assess factors for pCR. We also calculated the odds ratio (OR) with 95% confidence intervals (CI). All *p* values were two-sided in the tests, and *p* values ≤ 0.05 were considered statistically significant.

## 3. Results

### 3.1. Characteristics of Patients

Our study included 191 female patients with a median age of 48 years (range: 24–77). There were 108 (56.5%) patients in the non-obese group (BMI < 30 kg/m^2^) and 83 (43.5%) patients in the obese group (BMI ≥ 30 kg/m^2^). The median follow-up time was 39 months (range: 7.2–145.1). The baseline demographic and clinicopathological findings of the patients are outlined in Table 1. Median age was significantly higher in the obese patients compared to non-obese patients (54 vs. 44 years, *p* < 0.01). Obesity was more common in postmenopausal patients compared to premenopausal patients (60% vs. 40%, *p* < 0.01). The most common type of breast cancer was HR+/HER2− (43%), and the others were HR+/HER2+ (22%), HR−/HER2+ (10%), and TNBC (25%), respectively. There were 65 (79.3%) patients with Luminal-B and 17 (20.7%) patients with Luminal-A histological subtypes among the HR-positive/HER2-negative patient group. Invasive ductal carcinoma histology was observed in the majority of patients (84%). There were no significant differences in histological features (grade, Ki-67 score, ER/PR levels) and clinical staging of the tumor between non-obese and obese patients.

### 3.2. Treatment Interventions and Responses

In the HR-positive/HER2-negative group, anthracycline plus taxane-based regimens (n = 78, 95.2%), anthracycline-based regimens (n = 2, 2.4%), and taxane-based regimens (n = 2, 2.4%) had been used as neoadjuvant treatment. In the HER2-positive group, 41 of 62 patients (89.4%) received a combination chemotherapy with trastuzumab and pertuzumab, while 21 patients (10.6%) used chemotherapy with only trastuzumab. The neoadjuvant treatment regimens for the TNBC group were anthracycline plus taxane-based regimens (n = 23, 48.9%), antracycline and taxane-based regimen plus carboplatin (n = 21, 44.7%), anthracycline-based regimens (n = 2, 4.3%), and taxane-based regimens (n = 1, 2.1%). A total of 2 (4.3%) patients in the TNBC group received pembrolizumab with combination chemotherapy in a neoadjuvant setting. Approximately 25% of patients (45 of 181 patients) received dose-dense anthracycline among patients with treated anthracycline plus a taxane-based regimen in the entire patient cohort. Neoadjuvant treatment regimens were similar in non-obese and obese groups (*p* = 0.75).

Among the HR-positive/HER2-negative, HER2-positive, and TNBC groups, the pCR rates were 19.5%, 62.9%, and 40.4%, respectively. The pCR rates of patients in the obese group were significantly lower compared to those of non-obese patients (30% vs. 45%, *p* = 0.03). Treatment characteristics and pathological outcomes were shown in Table 2.

**Figure 1:**
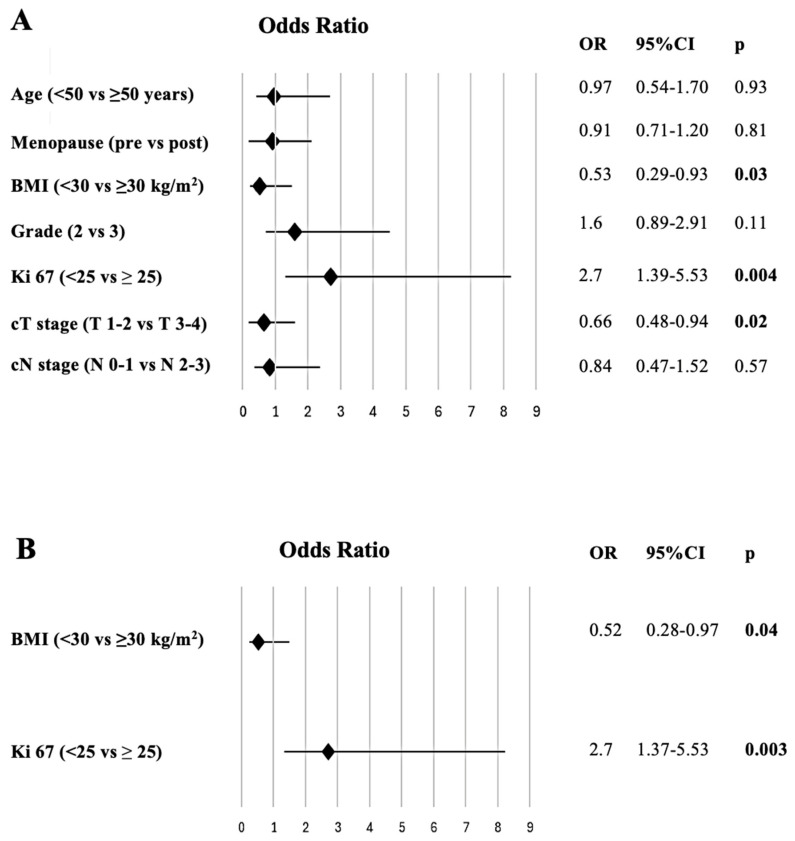
The forest plot shows the results of the univariate (**A**) and multivariate (**B**) analysis of factors for predicting pathological complete response.

The effects of BMI, age, menopause status, Ki 67 index, pathological grade, clinical T and N stage on pCR have been assessed by the logistic regression model. In the univariate analysis, body mass index (≥30 kg/m^2^ vs. <30 kg/m^2^), Ki-67 score (<25 vs. ≥25), and clinical T stage (T3–4 vs. T1–2) were significantly associated with a lower rate of pCR. In the multivariate analysis, obesity (OR: 0.52, 95%CI: 0.28–0.97; *p* = 0.04) and low Ki-67 score (HR: 2.7, 95%CI: 1.37–5.53; *p* = 0.003) were independently significantly associated with a decreased rate of pCR (Figure 1). The logistic regression model of factors affecting pCR is indicated in Table 3.

**Figure 2:**
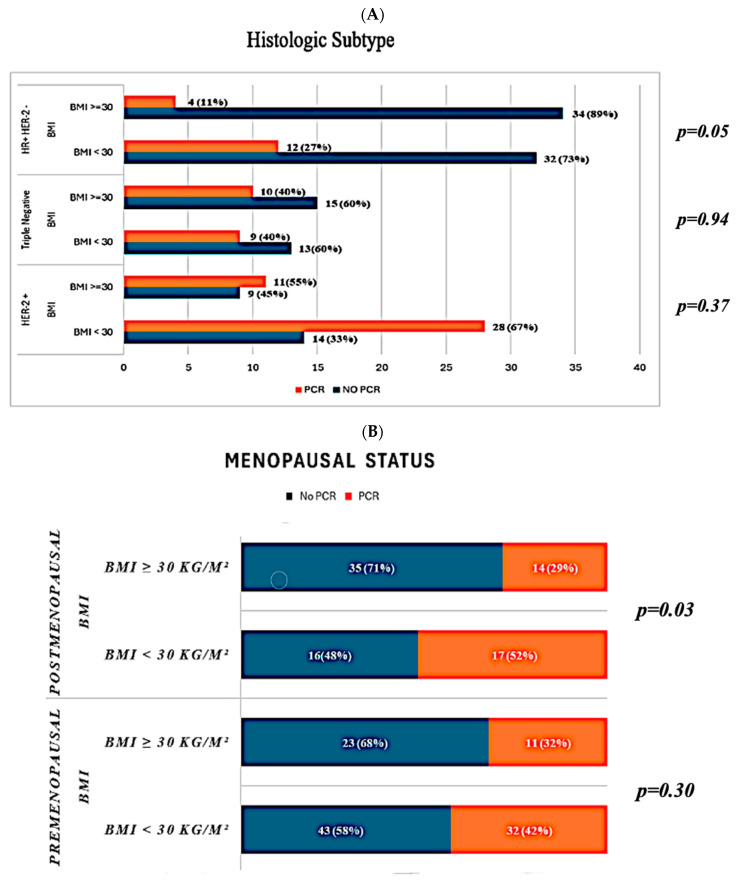
Pathological response rates based on histological subtypes (**A**) and menopausal status (**B**). BMI: body mass index; HR: hormone receptor; HER: human epidermal growth factor receptor; PCR: pathological complete response.

We investigated the impact of obesity on pCR by classifying breast cancer according to the histological subtype and menopausal status (Figure 2). Only in HR-positive/HER2-negative patients was the pCR rate statistically significantly lower in the obese group compared to the non-obese group (11% vs. 27%, *p* = 0.05). The HER2-positive (40% vs. 40%, *p* = 0.94) and TNBC (55% vs. 67%, *p* = 0.37) groups showed no statistically significant difference between obese and non-obese patient groups. Following a menopausal assessment, only postmenopausal patients showed a significant difference (29% vs. 52%, *p* = 0.03) in pCR rates between the obese and non-obese groups. In premenopausal patients, the rates of pCR were comparable across obese and non-obese groups (32% vs. 42%, *p* = 0.30).

## 4. Discussion

In the present, single-center retrospective study, we demonstrated that obesity was significantly associated with lower pCR rates in operable breast cancer patients following NACT. This association was observed independently of age, menopausal status, tumor grade, and clinical stage. We also demonstrated a statistically significant association, especially in postmenopausal women and the HR-positive/HER2-negative subgroup, as evidenced by higher pCR in the non-obese patient group compared to the obese group.

Obesity remains a significant global health concern, exacerbating the increasing incidence rates of breast cancer, which is the most prevalent cancer among women worldwide [2]. Numerous prospective studies and meta-analyses have indicated that higher body weight correlates with worse breast cancer outcomes [23,24]. Researchers have detected elevated levels of insulin resistance, pro-mitotic, and anti-apoptotic cytokines in obese patients compared to non-obese patients [25]. As a result, this may be a significant factor in breast cancer patients’ resistance to chemotherapy. On the other hand, elevated concentrations of chemotherapeutic drugs penetrating adipose tissue may decrease the requisite dosage needed to penetrate and disseminate inside tumor tissue, thereby affecting treatment efficacy. Moreover, to prevent chemotherapeutic overdosing and mitigate heightened toxicity, we can round the body surface area of most obese individuals to 2.0 square meters, regardless of accurate measurements. This may lead to an insufficient therapy dose.

NACT is currently the accepted standard of care for biologically high-risk breast cancer patients, and after NACT, achieving pCR provides a surrogate marker for improved long-term breast cancer outcomes [15]. The impact of high BMI on pCR in breast cancer patients undergoing NACT is a topic of uncertainty and controversy. Pooled analyses of 13 studies with a total of 18,702 women found that overweight and obese breast cancer patients had a much lower pCR rate with NACT than underweight or normal weight patients [16]. Fontanella et al. conducted a pooled analysis of clinical trials in Germany and found that normal weight patients had the best compliance to chemotherapy with positive outcomes, whereas patients with high BMI had a worse pCR rate with a detrimental impact on survival [18]. Similarly to our findings, several retrospective studies have shown that obesity negatively impacts pCR rates [26,27,28,29]. On the other hand, there are also studies in the literature that demonstrated that BMI had no impact on pCR [17,30,31]. Kogawa and colleagues demonstrated that keeping a normal weight during NACT is a prognostic indicator of poor survival, while it is not associated with pCR [32]. Thus, it may be crucial for patients to sustain a normal weight during NACT.

Obesity is well-documented to increase the risk of recurrence and mortality in hormone receptor-positive breast cancer patients [33,34]. Our investigation revealed that the assessment of the histological subtypes of breast cancer indicated a statistically significant correlation between obesity and lower pCR rates, particularly in HR-positive/HER2-negative patients. Similarly to our findings, an analysis of 8872 patients revealed a significant association between BMI and the pCR rate in HR-positive/HER2-negative patients but not in TNBC or HER2-positive patients [18]. The NeoALTTO trial’s exploratory analysis revealed that, for only the HR-positive patients group, being overweight or obese significantly reduced the chance of achieving a pCR rate, independently of other clinical characteristics such as planned surgery, nodal status, and tumor size (odds ratio [OR] = 0.55, 95%CI 0.30–1.01, compared to normal or underweight individuals; *p* = 0.053). However, HR-negative cases did not show any difference in the impact of BMI on pCR [35].

Several studies have documented an increase in breast cancer risk and poorer disease outcomes in postmenopausal women with a higher BMI [7,8,36]. In a study that evaluated the obesity and breast cancer risk for pre- and postmenopausal women among over six million Korean women, there was a positive relationship between obesity and breast cancer in postmenopausal women and an inverse association in premenopausal women [37]. In our study, we found that obesity was significantly more common in postmenopausal patients compared to premenopausal patients. Our investigation yielded that obesity is significantly associated with lower pCR rates in postmenopausal patients, not in premenopausal patients. Chen et al. [38] demonstrated that BMI had varied predictive values in pCR based on menopausal status, comparable to our study. Postmenopausal women showed a significant correlation with pCR (*p* = 0.004). However, in premenopausal women, there was no significant difference in pCR with respect to the BMI category. In another study, Kogawa et al. [39] also showed that an increase in BMI was not a significant predictor of pCR in premenopausal patients; however, it was strongly correlated with pCR in the postmenopausal cohort (*p* = 0.045).

The present study should be considered in light of several limitations. First of all, the single-center and retrospective design of the study can highlight potential selection biases and influencing factors that might affect the comprehension of the result and restrict the generalisability of the results. Secondly, due to our short follow-up period [39 months (range: 7.2–145.1 months)] and relatively low rate of recurrence (35 of 191 patients) and death (20 of 191 patients) in the last follow-up day, we could not analyze meaningful survival analysis to determine whether obesity impacts on overall survival (OS) and event-free survival (EFS) between pCR and non-pCR patients. Another limitation was that the study cohort encompassed a wide range of years (2012–2023), which included modifications to neoadjuvant treatment regimens.

## 5. Conclusions

According to our Turkish cohort of 191 patients with biologically high-risk breast cancer following NACT, we found evidence of obesity as an independently negative predictive factor of achieving pCR, especially in postmenopausal and HR-positive/HER2-negative patients. These findings suggest the importance of treating selected obese patients with a chemotherapy dosage based on actual weight. Longer follow-up and further work are needed to understand the role of obesity and breast cancer outcomes across all breast cancer subtypes. Investigating the mechanisms underlying a lower pCR rate in obese patients and developing prevention strategies may improve overall outcomes in obese breast cancer patients. Furthermore, the results of this study may increase awareness of modifiable lifestyle risk factors such as obesity.

## Figures and Tables

**Table 1:** Baseline demographic and clinicopathological findings.

Variables		All Patients n = 191 (%)	Body Mass Index (BMI) Status		
			BMI < 30 kg/m^2^ n = 108 (%)	BMI ≥ 30 kg/m^2^ n = 83 (%)	*p*
**Age (median) (years)**		48 (range: 24–77)	44 (range: 24–77)	54 (range: 30–74)	**<0.01**
**BMI (kg/m^2^) (median)**		28.6 (range 16.5–44.4)	25 (range: 16.5–29.8)	32.3 (range: 30–44.4)	**<0.01**
**Menopausal Status**	Premenopausal	109 (57)	75 (69)	34 (40)	**<0.01**
	Postmenopausal	82 (43)	33 (31)	49 (60)	
**Histology**	Ductal	161 (84)	93 (86)	68 (82)	0.24
	Lobular	5 (3)	4 (4)	1 (1)	
	Other	25 (13)	11 (10)	14 (17)	
**Subtype**	HR+, HER2−	82 (43)	44 (41)	38 (45)	0.15
	HR+, HER2+	42 (22)	28 (26)	14 (17)	
	HR−, HER2+	20 (10)	14 (13)	6 (7)	
	Triple negative	47 (25)	22 (20)	25 (30)	
**Grade**	Grade 1	1 (1)	1 (1)	0 (0)	0.5
	Grade 2	92 (48)	54 (50)	38 (45)	
	Grade 3	98 (51)	53 (49)	45 (55)	
**Ki-67(%) (median)**		25 (range: 5–80)	26 (range: 5–70)	25 (range: 5–80)	0.56
ER (%) (median) *		95 (range: 10–100)	95 (range: 10–100)	95 (range: 60–100)	0.48
PR (%) (median) *		75 (range: 0–100)	60 (range: 0–100)	80 (range: 0–100)	0.80
**cT Stage**	T1	18 (9)	11 (10)	7 (8)	0.45
	T2	113 (59)	68 (63)	45 (54)	
	T3	17 (8)	9 (9)	8 (9)	
	T4	43 (24)	20 (18)	23 (29)	
**cN Stage**	N0	21 (11)	13 (12)	8 (9)	0.15
	N1	80 (42)	38 (35)	42 (51)	
	N2	59 (31)	38 (35)	21 (26)	
	N3	31 (16)	19 (18)	12(14)	
**Stage**	Stage 1	15 (8)	11 (10)	4 (4)	0.71
	Stage 2	68 (36)	39 (36)	29 (36)	
	Stage 3	108 (56)	58(54)	50(60)	

* Only HR+/HER2-negative patients included. BMI: body mass index; HR: hormone receptor; HER: human epidermal growth factor receptor; ER: estrogen receptor; PR: progesterone receptor.

**Table 2:** Treatment characteristics and pathological outcomes.

Variables		All Patients n = 191 (%)	Body Mass Index (BMI) Status		
			BMI < 30 kg/m^2^ n = 108 (%)	BMI ≥ 30 kg/m^2^ n = 83 (%)	*p*
**Treatment**	Anthracycline plus Taxane based ± anti-HER2 agent	181 (94)	101 (94)	80 (96)	0.75
	Anthracycline	4 (2)	2 (2)	2 (3)	
	Taxane based ± anti-HER2 agent	6 (4)	5 (4)	1 (1)	
**Pathological Response**	Complete Response (pCR)	74 (39)	49 (45)	25 (30)	**0.03**
	No Complete Response	117 (61)	59 (55)	58 (70)	

BMI: body mass index; HER: human epidermal growth factor receptor.

**Table 3:** The logistic-regression model of factors affecting pathological complete response.

Variables		Pathological Response		Univariate Analysis		Multivariate Analysis	
		CR n = 74 (%)	Non-CR n = 117 (%)	OR (95%CI)	*p*	OR (95%CI)	*p*
**Age (years)**	<50	39 (39)	61 (61)	0.97 (0.54–1.70)	0.93		
	≥50	35 (38)	56 (62)				
**Menopausal Status**	Pre	43 (39)	66 (61)	0.91 (0.71–1.20)	0.81		
	Post	31 (38)	51 (62)				
**BMI (kg/m^2^)**	<30	49 (45)	59 (55)	0.53 (0.29–0.98)	**0.03**	0.52 (0.28–0.97)	**0.04**
	≥30	25 (30)	58 (70)				
**Grade**	Grade 2	30 (33)	62 (67)	1.6 (0.89–2.91)	0.11		
	Grade 3	43 (44)	55 (56)				
**Ki 67**	<25	14 (23)	46 (77)	2.7 (1.39–5.53)	**0.004**	2.7 (1.37–5.53)	**0.003**
	≥25	60 (46)	71 (54)				
**cT Stage**	T1–2	58 (44)	73 (66)	0.66 (0.48–0.94)	**0.02**		
	T3–4	16 (27)	44 (73)				
**cN Stage**	N0–N1	41 (40)	60 (60)	0.84 (0.47–1.52)	0.57		
	N2–N3	33 (37)	57 (63)				

BMI: body mass index; CR: complete response; OR: odds ratio.

## Data Availability

The datasets generated and/or analyzed during the current study are available from the corresponding author.
